# Combination Therapy of RAS Inhibition and SGLT2 Inhibitors Decreases Levels of Endotrophin in Persons with Type 2 Diabetes

**DOI:** 10.3390/biomedicines11113084

**Published:** 2023-11-17

**Authors:** Alexandra Louise Møller, Stefanie Thöni, Felix Keller, Samir Sharifli, Daniel Guldager Kring Rasmussen, Federica Genovese, Morten Asser Karsdal, Gert Mayer

**Affiliations:** 1 Nordic Bioscience, Herlev Hovedgade 205-207, 2730 Herlev, Denmark; 2 Department of Biomedical Sciences, Faculty of Health and Medical Sciences, University of Copenhagen, 2200 Copenhagen, Denmark; 3 Department of Internal Medicine IV (Nephrology and Hypertension), Medical University Innsbruck, 6020 Innsbruck, Austria

**Keywords:** diabetic kidney disease, biomarker, fibrosis, extracellular matrix, collagen, endotrophin

## Abstract

We investigated for the first time the effect of combination therapy of renin–angiotensin system inhibition (RASi) and sodium–glucose co-transporter-2 inhibitors (SGLT2is) on endotrophin (ETP), a pro-fibrotic signaling molecule reflecting collagen type VI formation, measured in the plasma of persons with type 2 diabetes (T2D). ETP was measured using the PRO-C6 ELISA in 294 individuals from the “Drug combinations for rewriting trajectories of renal pathologies in type 2 diabetes” (DC-ren) project. In the DC-ren study, kidney disease progression was defined as a >10% decline in the estimated glomerular filtration rate (eGFR) to an eGFR < 60 mL/min/1.73 m^2^. Among the investigated circulating markers, ETP was the most significant predictor of future eGFR. Combination therapy of RASi and SGLT2is led to a significant reduction in ETP levels compared to RASi monotherapy (*p* for slope difference = 0.002). Higher levels of baseline plasma ETP were associated with a significantly increased risk of kidney disease progression (*p* = 0.007). In conclusion, plasma ETP identified individuals at higher risk of kidney disease progression. The observed decreased levels of plasma ETP with combination therapy of RASi and SGLT2is in persons with T2D may reflect a reduced risk of kidney disease progression following treatment with SGLT2is.

## 1. Introduction

Diabetic kidney disease (DKD) is a severe complication of diabetes, often accompanied by cardiovascular disease. DKD occurs in up to 40% of persons with type 2 diabetes (T2D) and represents the leading cause of chronic kidney disease (CKD) and kidney failure [1]. The prevalence of DKD is expected to increase in parallel with the global rise in diabetes, which is projected to increase to 784 million by 2045 [2].

The mechanisms of DKD include a series of disturbed metabolic, hemodynamic, inflammatory, and fibrotic processes [3,4,5] initiated by hyperglycemia. Renin–angiotensin system inhibition (RASi) has been the cornerstone for managing DKD for decades. Treatment with an angiotensin-converting enzyme inhibitor (ACEi) or angiotensin receptor blocker (ARB) is recommended for all persons with diabetes and hypertension who have moderately increased albuminuria (urinary albumin-to-creatinine ratio; UACR 30–299 mg/g creatinine) and is strongly recommended for those with an estimated glomerular filtration rate (eGFR) < 60 mL/min/1.73 m^2^ and/or severely increased albuminuria (UACR ≥ 300 mg/g creatinine) [6]. In recent years, sodium–glucose co-transporter-2 inhibitors (SGLT2is) have demonstrated beneficial cardiorenal protective effects and are now recommended as first-line treatment for DKD in addition to RASi. SGLT2is are currently recommended for individuals with T2D and an eGFR > 20 mL/min/1.73 m^2^, independent of glycemic control. This recommendation is based on evidence demonstrating their effectiveness in reducing DKD progression, risk of kidney failure, and cardiovascular events [6,7,8,9]. 

Despite recent therapeutic advances, the risk of developing DKD remains high, and the early stages of the disease and disease progression may go unnoticed until the manifestation of serious complications. Novel biomarkers are urgently needed to improve the health outcomes for these patients [10]. Linking the mechanisms involved in disease progression to therapeutic strategies is central to finding targeted interventions that reduce the progression of DKD [11]. Likewise, novel biomarkers to identify persons at increased risk of progression and for predicting response to treatment are of major importance to develop the concept of precision medicine. 

Fibrosis, characterized by extensive deposition of extracellular matrix (ECM) components [12], is considered the final common pathway for loss of kidney function in DKD [13]. As the turnover of ECM proteins, such as collagens, is closely linked with the development of kidney fibrosis [14], assessment of biomarkers of ECM turnover may identify persons with ongoing pro-fibrotic activity who are at increased risk of DKD progression. 

Interestingly, collagens are emerging as more than passive structural proteins, as proteolytic-derived fragments from collagens can have vital signaling functions [15]. 

Endotrophin (ETP), a signaling fragment generated during the formation of collagen type VI, has been shown to possess pro-inflammatory and pro-fibrotic properties [16,17,18]. ETP is released during collagen type VI formation when the C-terminal pro-peptide of the α3 chain (C5 domain) is cleaved off from the mature molecule [19]. The C5 domain has been shown to undergo proteolysis via the extracellular metalloproteinase bone morphogenic protein-1 (BMP-1), releasing ETP [20]. 

In previous studies [21,22,23], circulating ETP, measured using the PRO-C6 ELISA [24], was associated with kidney and cardiovascular complications and mortality in persons with T2D. 

The aim of this study was to investigate for the first time the effect of combination therapy of RASi and SGLT2is on plasma ETP in persons with DKD. 

## 2. Materials and Methods

### 2.1. Study Population

This study is based on participants included in the “Drug combinations for rewriting trajectories of renal pathologies in type 2 diabetes” project (DC-ren.eu; Horizon 2020, proposal SEP-210574920). DC-ren aims to develop a decision support software application for optimizing drug combination therapy in persons with DKD. For this purpose, DC-ren focuses on biomarkers to allow the prediction of changes in kidney function in response to a specific drug combination therapy. Using annual assessments of a panel of biomarkers, participants were stratified into different pathophysiological states of DKD. As the underlying pathophysiology of each state also represents the point of action of treatment, the state-specific drug response can be deduced, ultimately allowing statements on optimal drug combinations on a personalized level. Detailed information on the DC-ren study is presented elsewhere [25].

To develop this biomarker-based algorithm, DC-ren relies on data and samples from 456 selected participants of the Prospective Cohort Study in Patients with Type 2 Diabetes Mellitus for Validation of Biomarkers (PROVALID) study. Detailed information on the study design and baseline characteristics has been described previously [26]. In brief, PROVALID recruited about 4000 persons with T2D in five European countries. PROVALID provides repetitive annual information on laboratory measurements and medication and data on the incidence and progression of kidney and cardiovascular disease. In addition, annual blood and urine samples for the validation of biomarkers were collected and stored. PROVALID was approved in each participating country by the responsible local Institutional Review Board. All study participants signed an informed consent. 

DC-ren only includes participants for which annual clinical and laboratory follow-up data and samples are available. Individuals on continuous treatment with RASi with at least one follow-up were selected. A “background” population treated with RASi monotherapy and a “drop-in” population, in which either a SGLT2i, glucagon-like peptide 1 receptor agonist, or mineralocorticoid receptor antagonist (MRA) was initiated on top of RASi, were defined. Given the intention of DC-ren, participants were selected based on pre-specified annual eGFR trajectories, reflecting the high intra-individual variability of longitudinal eGFR in DKD. Trajectories were defined based on different sequences of “controlled” and “uncontrolled disease”. Uncontrolled disease was defined as an annual decrease in eGFR >10%, while controlled disease was defined as an increase or decrease in eGFR of <5%. 

In the DC-ren study, the endpoint for kidney disease progression was defined as a decline in eGFR >10% from baseline to an eGFR < 60 mL/min/1.73 m^2^. This cut-off is based on the finding that the biological variation of eGFR is 12.5% [27]. As stated by Kidney Disease: Improving Global Outcomes (KDIGO), there is considerable controversy about what constitutes a significant change in eGFR [28]. In contrast to conventional thresholds (i.e., 30% or 40%), where irreversible kidney damage has occurred, we are interested in early detection of kidney function decline while still considering random fluctuations in the eGFR. Ethical approval of the present study was granted by the Innsbruck Medical University Ethics Committee (number 1188/2020). The present study focuses on the participants treated with RASi monotherapy or a combination of RASi and SGLT2is.

### 2.2. Measurement of Endotrophin Using ELISA

Levels of ETP were measured using the PRO-C6 ELISA (Nordic Bioscience, Herlev, Denmark) in plasma from 294 persons with T2D enrolled in the DC-ren study, treated using either RASi monotherapy or combination therapy of RASi and SGLT2is. The assay was carried out as previously described [24]. Due to either an insufficient sample amount, hemolytic or lipemic samples, or inaccurate test results, the baseline levels of plasma ETP were not available for 55 participants.

### 2.3. Statistics

Normally and nonnormally distributed variables are presented as mean ± standard deviation (SD) or median with interquartile range (IQR), respectively. Categorical variables are shown as total numbers with corresponding percentages. Differences in baseline characteristics between persons with plasma ETP levels above and below the median were assessed using a *t*-test for normally distributed variables, a Kruskal–Wallis test for nonnormally distributed variables, and an χ^2^ test for categorical variables. 

A multivariate Least Absolute Shrinkage and Selection Operator (LASSO) regression analysis was used to assess the prognostic power of ETP for future eGFR and UACR, respectively. By applying nested cross-validation, we assessed the mean prognostic importance of each employed biomarker. The circulating markers included were plasma ETP, hemoglobin, serum potassium, high-density lipoprotein (HDL)-cholesterol, low-density lipoprotein (LDL)-cholesterol, total cholesterol, serum triglycerides, diastolic blood pressure, systolic blood pressure, HbA1c, serum albumin, blood glucose, and C-reactive protein. 

To assess the effect of RASi and SGLT2i treatment on plasma ETP, longitudinal plasma ETP levels stratified by treatment group over time were analyzed using linear regression. Differences between the group slopes were assessed using a *t*-test. 

The Kaplan–Meier estimator was applied to compare the risks of experiencing the kidney endpoint (>10% decline in eGFR to an eGFR < 60 mL/min/1.73 m^2^) according to the baseline ETP levels split by the median. Differences between the groups were assessed using a log-rank test. The Kaplan–Meier estimator is based on participants with an available baseline plasma ETP, whereas all other analyses are based on the total study cohort. All two-tailed *p* < 0.05 were considered significant. Statistical analyses were performed using R (version 4.2.1).

## 3. Results

Of the 294 participants included in the present study, 102 (35%) received combination therapy of RASi and SGLT2is during follow-up. The number of follow-ups ranged from one to five, with the majority of the participants (71%) having three annual follow-ups. The total study cohort consisted of 144 (49%) females, mean age was 65 ± 9 years, diabetes duration was 14 ± 8 years, and the medians (IQR) of the eGFR and UACR were 68 (54–78) mL/min/1.73 m^2^ and 10 (4.4–28) mg/g, respectively. The clinical characteristics of the overall study cohort and of the participants with missing plasma ETP information at baseline are presented in Table 1.

Baseline plasma ETP levels were available for 239 (81%) of the total study cohort. The mean baseline plasma ETP was 12.2 ± 5 ng/mL. The cohort with available baseline ETP levels consisted of 120 (50%) females, mean age was 66 ± 8 years, diabetes duration was 14 ± 8 years, and the medians (IQR) of the eGFR and UACR were 66 (52–78) mL/min/1.73 m^2^ and 11 (4.7–30) mg/g, respectively. The clinical characteristics of the study cohort with available baseline ETP levels and stratified by the median baseline ETP are presented in Table 2. Participants with higher levels of ETP had a lower eGFR (*p* < 0.001), a higher UACR (*p* = 0.03), and lower hemoglobin (*p* = 0.01) (Table 2). No differences were seen for other clinical variables when the baseline ETP levels were split by the median.

**Figure 1:**
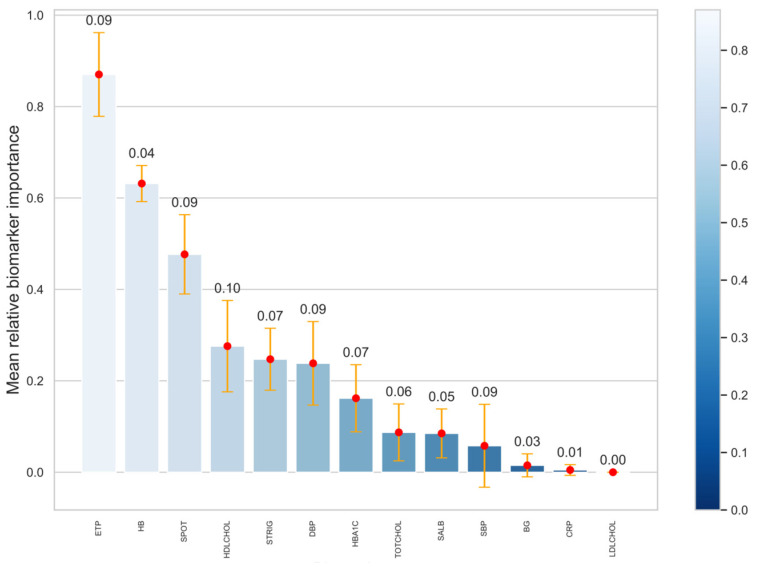
Plasma ETP as a prognostic risk marker for kidney function decline compared to other clinical variables. The plot shows the average significance of the investigated biomarkers for future eGFR assessed using a LASSO model with applied nested cross-validation. The horizontal axis indicates the biomarkers employed. The vertical axis displays the average significance. The height of the bars represents average mean significance along with corresponding standard deviation (error bars). The plot is organized into a sorted fashion; from left to right is the highest to the lowest feature importance. ETP: endotrophin; HB: hemoglobin; SPOT: serum potassium; HDLCHOL: HDL cholesterol; LDLCHOL: LDL cholesterol; TOTCHOL: total cholesterol; STRIG: serum triglycerides; DBP: diastolic blood pressure; SBP: systolic blood pressure; SALB: serum albumin; BG: blood glucose; CRP: C-reactive protein.

The ability of ETP to predict the future eGFR compared to other clinical variables is shown in Figure 1. Interestingly, plasma ETP was the most significant predictor of eGFR, followed by hemoglobin (Figure 1). Plasma ETP had a low predictive value for future UACR. However, none of the investigated markers demonstrated a high predictive value for future UACR.

Change in the levels of traditional clinical variables by treatment group is shown in Table 3. Treatment with RASi and SGLT2is significantly decreased the levels of HbA1c (*p* < 0.001), BMI (*p* < 0.001), serum potassium levels (*p* = 0.05), and systolic and diastolic blood pressure (*p* = 0.02 and *p* = 0.02, respectively), and increased the levels of hemoglobin (*p* = 0.001) compared to RASi monotherapy (Table 3). Moreover, the combination of RASi and SGLT2i therapy trended toward a slower rate of decline in eGFR (*p* = 0.074).

**Figure 2:**
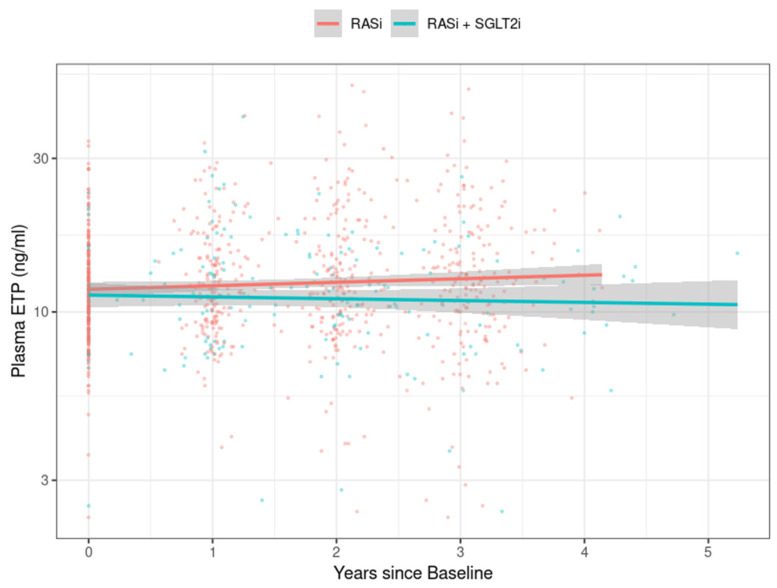
Levels of plasma ETP decreased with a combination therapy of RASi and SGLT2is compared to RASi monotherapy. Longitudinal plasma endotrophin (ETP) levels stratified by treatment group over time (*p* for slope difference = 0.002). RASi: renin–angiotensin system inhibitor; SGLT2i: sodium–glucose co-transporter 2 inhibitor.

The longitudinal plasma ETP levels stratified by treatment group over time are shown in Figure 2. The levels of plasma ETP were significantly lower in persons treated with SGLT2is in combination with RASi compared to RASi monotherapy (*p* for slope difference = 0.002).

**Figure 3:**
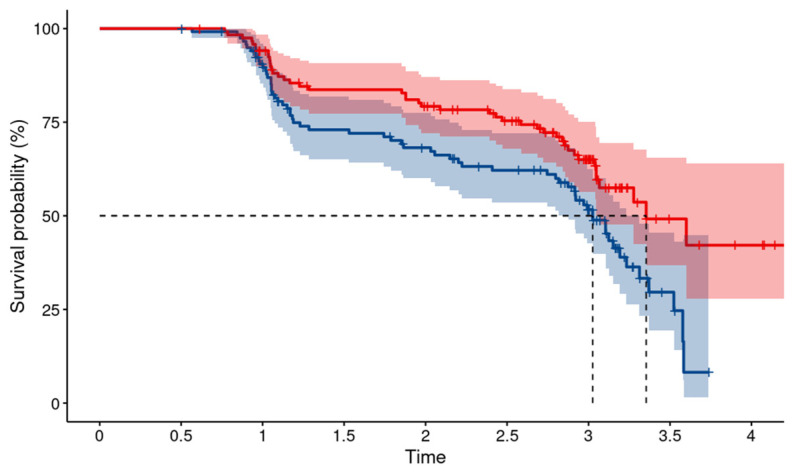
Higher levels of plasma ETP at baseline are associated with kidney function decline. Kaplan–Meier estimates for baseline plasma endotrophin (ETP) stratified by the median for the kidney endpoint defined as a >10% decline in eGFR from baseline to an eGFR < 60 mL/min/1.73 m^2^ (*p* = 0.007). Time is indicated in years. Blue line, baseline ETP above the median; red line, baseline ETP below the median.

Higher levels of baseline plasma ETP were associated with a higher risk of kidney disease progression, defined as a >10% decline in eGFR from baseline to an eGFR < 60 mL/min/1.73 m^2^ over time (*p* = 0.007) (Figure 3).

## 4. Discussion

In this study, higher baseline levels of plasma ETP were associated with a lower eGFR and higher UACR, respectively. These findings are in line with previous studies, where circulating levels of ETP were associated with kidney disease severity in persons with T2D [21,22,23]. Importantly, previous studies have shown that collagen type VI is accumulated in the kidneys of persons with kidney disease [29,30,31]. Furthermore, histological investigations have confirmed the co-localization of ETP with collagen type VI in fibrotic kidneys [32].

A significant association between plasma ETP and future eGFR was found, indicating that ETP is a prognostic risk marker for kidney function decline. Interestingly, ETP was the biomarker with the highest predictive value for eGFR compared to other investigated traditional clinical variables. We have previously shown that circulating ETP is an independent risk marker of kidney function decline and development of kidney failure in persons with T2D [21,22,23] after adjustment for conventional risk factors. Together, these findings underline that circulating ETP is a relevant risk marker for CKD progression and the development or progression of kidney complications in diabetes.

In this study, plasma ETP was not a risk marker for future UACR; however, due to the high variability between measurements of UACR, UACR is generally hard to predict [33,34]. Nevertheless, we have previously shown that higher levels of circulating and urinary ETP, respectively, were independently associated with albuminuria progression in persons with T2D [23]. Albuminuria is linked to DKD progression but lacks sensitivity, as many persons with T2D and kidney disease progression do not have albuminuria [35,36].

In addition to the beneficial effects observed on traditional clinical variables, the levels of plasma ETP were significantly lower in persons treated with RASi and SGLT2is compared to RASi monotherapy. In the RASi and SGLT2i group, the increase in plasma ETP was less pronounced over time, accompanied by a slower decline in eGFR, indicating that SGLT2is may affect collagen type VI formation and the levels of ETP. These data suggest that the ETP biomarker, reflecting fibrogenesis and pro-fibrotic signaling, has the potential to be a pharmacodynamic marker of response to SGLT2is and may be used to monitor the efficacy of treatment in slowing the progression of kidney function decline.

The potential anti-fibrotic effect of SGLT2is has been suggested before [6,37,38,39,40] and could be one of the mechanisms for the beneficial effects observed in persons with CKD treated with these drugs. In persons with T2D, treatment with canagliflozin decreased the plasma levels of inflammatory and fibrosis biomarkers, including kidney injury molecule-1 (KIM-1) and tumor necrosis factor receptors TNFR-1 and TNFR-2, when compared to placebo [39]. Early decreases in TNFR-1 and TNFR-2 during canagliflozin treatment were independently associated with a lower risk of DKD progression, suggesting that TNFR-1 and TNFR-2 may also serve as potential pharmacodynamic markers of the response to SGLT2is. In persons with T2D with inadequate glycemic control despite treatment with metformin, a reduction in plasma levels of TNFR1, interleukin 6 (IL-6), matrix metalloproteinase 7 (MMP7), and fibronectin 1 (FN1) was observed in individuals treated with canagliflozin compared to glimepiride [6]. In persons with T2D, treatment with dapagliflozin decreased the urinary excretion of KIM-1 and IL-6 compared to placebo [40]. During dapagliflozin treatment, changes in albuminuria correlated with changes in KIM-1, indicating that the albuminuria-lowering effect of dapagliflozin therapy may be the result of decreased intraglomerular pressure or reduced tubular cell injury [40]. Moreover, treatment with empagliflozin reduced the levels of factors implicated in inflammation and tissue fibrosis in experimental models of diabetes [41,42]. Overall, these data suggest that SGLT2is contribute to the reversal of molecular processes related to inflammation, ECM turnover, and fibrosis.

Although the mechanism of action through which SGLT2is exert kidney-protective effects are yet to be fully elucidated, a reduction in glomerular hyperfiltration and the normalization of glomerular hemodynamics via the activation of tubuloglomerular feedback are thought to be some of them [43]. However, even with the addition of SGLT2is to ACEi or ARBs, considerable residual risks associated with DKD remain [44]. Given that a number of persons with DKD continue to progress despite treatment, possibly due to the fact that the T2D population is heterogeneous in its demographics, clinical features, and prognosis [45], there is a current need for additional therapies and more personalized treatment.

The pharmacodynamic potential of ETP in persons with T2D has been investigated in previous studies [22,46]. In the AWARD-7 trial, including persons with T2D and moderate-to-severe CKD, dulaglutide treatment decreased levels of circulating ETP compared to insulin glargine [46]. This indicates that dulaglutide treatment may reduce interstitial fibrosis by lowering collagen type VI formation and levels of ETP. Plasma ETP was not impacted by canagliflozin treatment in the CANVAS trial, where the total population had an average eGFR of 77 ± 19 mL/min/1.73 m^2^ and was predominantly classified as having a normal to mildly increased UACR (72.5%) [22]. The lack of impact of canagliflozin on plasma ETP may be due to participants having a generally milder disease and potentially lower disease activity than the DC-ren study population. Nevertheless, in the CANVAS trial, ETP was an independent risk marker for all investigated outcomes [22].

As circulating ETP identifies persons at increased risk of experiencing clinically relevant kidney and cardiovascular outcomes, this biomarker may be used to select persons who could benefit from preventive treatment and for patient enrichment in future clinical trials.

Several individual circulating and urinary biomarkers are associated with DKD progression, with some of the most reported being markers of inflammation and fibrosis. These include tumor necrosis factor-alpha (TNF-α), C-reactive protein (CRP), fibroblast growth factors-21 and 23, transforming growth factor-beta (TGF-β), neutrophil gelatinase-associated lipocalin (NGAL), and KIM-1 [47]. However, there are strong correlations between many of these biomarkers [48]. Despite efforts to develop novel prognostic biomarkers for DKD, few trials use biomarkers other than eGFR and albuminuria as stratification variables, entry criteria, or surrogate outcome measures. An exception was the PRIORITY trial, in which CKD273 (a urinary biomarker pattern for fibrosis, including collagen fragments), was used to stratify participants into a drug vs. placebo arm [49].

If validated biomarkers of fibrosis, reflecting a pathophysiological mechanism involved in the progression of DKD, are implemented in clinical trials investigating agents with potential anti-fibrotic effects, these may ultimately guide treatment decision in clinical practice. In this study, plasma ETP was a pharmacodynamic biomarker, and it may act as a tool to monitor the potential impact of therapies on fibrogenesis (or at least collagen type VI formation) and to reduce levels of a potentially deleterious signaling fragment.

To date, no human intervention studies targeting ETP have been conducted. Nevertheless, it has been suggested that neutralizing the pro-fibrotic ETP using antibodies can slow the unbalanced ECM turnover in fibrogenesis [50]. Interestingly, recent data from a CKD model showed that ETP neutralization using targeted antibody treatment protected against kidney fibrosis [51]. These data suggest that neutralizing ETP holds promise as a therapeutic approach for intervening with kidney fibrosis in CKD.

The nonsteroidal MRA finerenone, which reduces kidney and cardiac fibrosis in experimental studies [52,53], has been shown to reduce the progression of kidney and cardiovascular outcomes in persons with T2D [54]. Thus, ETP may be used to select individuals who could benefit from preventive treatment with finerenone.

The strength of this study was the availability of annual clinical and laboratory follow-up data; however, a complete follow-up was not available for all participants. A limitation of this study was the few observations regarding the incidence of outcomes; therefore, the association of plasma ETP and incidence of cardiovascular complications and mortality was not investigated. Nevertheless, the association of baseline plasma ETP and kidney and cardiovascular outcomes and mortality is currently being investigated in about 3100 participants from the PROVALID study.

## 5. Conclusions

In conclusion, plasma ETP decreased with combination therapy of RASi and SGLT2is compared to RASi monotherapy, indicating that SGLT2is may affect collagen type VI formation and the levels of ETP. Higher ETP levels at baseline were associated with a significantly higher risk of kidney function decline, indicating that ETP identifies persons with high disease activity where the potential of anti-fibrotic treatment becomes more prominent. The ETP biomarker, reflecting fibrogenesis, may be used to monitor the efficacy of treatment in slowing the progression of kidney function decline.

Further research is needed to elucidate the mechanisms of action of current glucose-lowering therapies in reducing kidney fibrosis. Future studies are required to evaluate the potential of circulating ETP to predict a clinically meaningful response to treatment with these agents; therefore, the effects of SGLT2is and MRAs on the circulating levels of ETP are currently under investigation in post hoc analyses from clinical trials, including persons with T2D. In the future, the goal is to have the ETP biomarker FDA-approved as a tool to enrich or stratify clinical trials based on the risk of outcome. The Letter of Support received from the FDA [55], encouraging further investigation of ETP as a prognostic biomarker for the enrolment of participants in heart failure with preserved ejection fraction trials, is a step in this direction.

## Figures and Tables

**Table 1:** Baseline clinical characteristics of the overall study cohort and participants with missing ETP.

	All (*n* = 294)	Missing ETP (*n* = 55)
RASi	192 (65)	3 (5.5)
RASi + SGLT2i	102 (35)	52 (95)
Age (years)	65 ± 9	61 ± 10
Female sex	144 (49)	24 (44)
BMI (kg/m^2^)	32 ± 5	34 ± 6
Current or former smoker	132 (45)	23 (42)
Systolic BP (mmHg)	138 ± 16	140 ± 14
Diastolic BP (mmHg)	78 ± 9.5	79 ± 8.8
Plasma ETP (ng/mL)	12.2 ± 5.0	
eGFR (mL/min/1.73 m^2^)	68 (54–78)	69 (61–83)
UACR (mg/g)	10 (4.4–28)	7.0 (3.6–24)
Diabetes duration (years)	14 ± 8	14 ± 6
HbA1c (%)	7.5 ± 1.4	8.4 ± 5.8
C-reactive protein (mg/dL)	0.3 (0.1–0.6)	0.3 (0.1–0.7)
Hemoglobin (g/dL)	13.7 ± 1.5	13.9 ± 1.8
LDL cholesterol (mg/dL)	98 ± 38	89 ± 36
HDL cholesterol (mg/dL)	49 ± 14	43 ± 9.3
Serum triglycerides (mg/dL)	157 (106–213)	182 (133–246)
Serum albumin (g/dL)	4.5 (4.2–4.8)	4.6 (4.3–4.9)
Serum potassium (mmol/L)	4.4 (4.1–4.7)	4.4 (4.2–4.7)

Data are *n* (%), mean ± SD, or median (IQR). ETP: endotrophin; RASi: renin–angiotensin system inhibitor; SGLT2i: sodium–glucose co-transporter 2 inhibitor; BP: blood pressure; UACR: urinary albumin-to-creatinine ratio.

**Table 2:** Baseline clinical characteristics of the study cohort stratified by median ETP levels.

	All (*n* = 239)	ETP < Median (*n* = 120)	ETP > Median (*n* = 119)	*p*
RASi	189 (79)	92 (77)	97 (82)	
RASi + SGLT2i	50 (21)	28 (23)	22 (19)	
Age (years)	66 ± 8	65 ± 7	67 ± 10	0.13
Female sex	120 (50)	65 (54)	55 (46)	0.27
BMI (kg/m^2^)	31 ± 5	30 ± 4	31 ± 5	0.11
Current or former smoker	109 (46)	54 (45)	55 (46)	0.70
Systolic BP (mmHg)	137 ± 16	138 ± 18	136 ± 13	0.23
Diastolic BP (mmHg)	78 ± 10	78 ± 10	78 ± 9	0.83
Plasma ETP (ng/mL)	12.2 ± 5.0	8.7 ± 1.7	15.8 ± 4.6	<0.001
eGFR (mL/min/1.73 m^2^)	66 (52–78)	74 (63–81)	59 (45–72)	<0.001
UACR (mg/g)	11 (4.7–30)	8 (4.4–23)	15 (5.3–39)	0.03
Diabetes duration (years)	14 ± 8	13 ± 8	14 ± 8	0.32
HbA1c (%)	7.3 ± 1.2	7.3 ± 1.3	7.2 ± 1.1	0.43
C-reactive protein (mg/dL)	0.3 (0.1–0.5)	0.3 (0.1–0.5)	0.2 (0.1–0.5)	0.45
Hemoglobin (g/dL)	13.6 ± 1.4	13.9 ± 1.4	13.4 ± 1.5	0.01
LDL cholesterol (mg/dL)	100 ± 38	102 ± 41	97 ± 36	0.30
HDL cholesterol (mg/dL)	50 ± 15	52 ± 15	48 ± 15	0.08
Serum triglycerides (mg/dL)	142 (100–204)	142 (98–195)	151 (104–213)	0.41
Serum albumin (g/dL)	4.5 (4.2–4.8)	4.5 (4.2–4.8)	4.5 (4.2–4.8)	0.45
Serum potassium (mmol/L)	4.4 (4.1–4.7)	4.3 (4.1–4.6)	4.5 (4.1–4.7)	0.14

Data are *n* (%), mean ± SD, or median (IQR). ETP: endotrophin; RASi: renin–angiotensin system inhibitor; SGLT2i: sodium–glucose co-transporter 2 inhibitor; BP: blood pressure; UACR: urinary albumin-to-creatinine ratio.

**Table 3:** Change in clinical variables following treatment with RASi monotherapy vs. combination therapy with RASi and SGLT2i.

	RASi	RASi + SGLT2i	*p*
eGFR (mL/min/1.73 m^2^)	−1.96 ± 12	−0.27 ± 10	0.07
UACR (mg/g)	2.51 ± 165	−7.56 ± 121	0.44
Serum albumin (g/dL)	−0.04 ± 0.3	0.00 ± 0.4	0.16
Hemoglobin (g/dL)	−0.06 ± 1.2	0.32 ± 1.6	0.001
Serum potassium (mmol/L)	0.06 ± 0.5	−0.02 ± 0.4	0.05
Serum triglycerides (mg/dL)	4.36 ± 125	0.54 ± 113	0.71
BMI (kg/m^2^)	0.00 ± 1.5	−0.53 ± 2.2	<0.001
Systolic BP (mmHg)	0.05 ± 16.7	−3.19 ± 14.5	0.02
Diastolic BP (mmHg)	0.06 ± 9.7	−1.74 ± 8.8	0.02
HbA1c (%)	0.13 ± 1.0	−0.24 ± 1.4	<0.001
C-reactive protein (mg/dL)	0.01 ± 2.6	0.09 ± 2.1	0.72
LDL cholesterol (mg/dL)	−1.72 ± 32.6	−0.68 ± 40.7	0.77
HDL cholesterol (mg/dL)	−0.36 ± 10.1	0.91 ± 8.2	0.12

Data are mean ± SD. UACR: urinary albumin-to-creatinine ratio; BP: blood pressure; RASi: renin–angiotensin system inhibitor; SGLT2i: sodium–glucose co-transporter 2 inhibitor.

## Data Availability

Data is available upon reasonable request to the corresponding author.
